# STEAP2 Knockdown Reduces the Invasive Potential of Prostate Cancer Cells

**DOI:** 10.1038/s41598-018-24655-x

**Published:** 2018-04-19

**Authors:** Stephanie E. A. Burnell, Samantha Spencer-Harty, Suzie Howarth, Owen Bodger, Howard Kynaston, Claire Morgan, Shareen H. Doak

**Affiliations:** 1 Institute of Life Science, Swansea University Medical School, Singleton Park, Swansea, SA2 8PP Wales UK; 2 Cellular Pathology, Abertawe Bro Morgannwg University Health Board, Singleton Hospital, Sketty Lane, Sketty, Swansea, SA2 8QA, Wales, UK; 3 Histopathology, Abertawe Bro Morgannwg University Health Board, Morriston Hospital, Heol Maes Eglwys, Morriston, Swansea, SA6 6NL, Wales, UK; 4 Cardiff School of Medicine, Cardiff University, Heath Park, Cardiff, CF14 4XN Wales UK

## Abstract

Six-transmembrane epithelial antigen of the prostate-2 (STEAP2) expression is increased in prostate cancer when compared to normal prostate, suggesting STEAP2 may drive prostate cancer progression. This study aimed to establish the functional role of STEAP2 in prostate tumourigenesis and evaluate if its knockdown resulted in reduced invasive potential of prostate cancer cells. PC3 and LNCaP cells were transfected with STEAP2 siRNA and proliferation, migration, invasion and gene expression analyses were performed. STEAP2 immunohistochemistry was applied to assess the protein expression and localisation according to Gleason score in 164 prostate cancer patients. Invasion significantly decreased in both cell lines following STEAP2 knockdown. PC3 proliferation and migration capacity significantly reduced, while LNCaP cell morphology and growth characteristics were altered. Additionally, STEAP2 downstream targets associated with driving invasion were identified as *MMP3, MMP10, MMP13, FGFR4, IL1β, KiSS1* and *SERPINE1* in PC3 cells and, *MMP7* in LNCaP cells, with *CD82* altered in both. In patient tissues, STEAP2 expression was significantly increased in prostate cancer samples and this significantly correlated with Gleason score. These data demonstrate that STEAP2 drives aggressive prostate cancer traits by promoting proliferation, migration and invasion and significantly influencing the transcriptional profile of ten genes underlying the metastatic cascade.

## Introduction

Prostate cancer (PCa) is the second most common cancer worldwide, with one in eight men being diagnosed in the UK and one in five/six in the USA^1,2^. There is currently no routine PCa screening system, however, according to the 2016 American Cancer Society guideline, from the age of 45, men should receive information and be allowed to make their own decision regarding screening, but this only occurs if a patient has specific risk factors or presents with urinary/sexual symptoms^3^. Men without symptoms are discouraged from PCa screening by the US Preventive Services Task Force due to the risk of detecting slow growing cancers that will not require treatment within the patients’ lifetime^4^. As slow growing cancers cannot be distinguished from fast growing, aggressive cancers, new prognostic biomarkers are required to improve patient stratification, assist with clinical management of the disease and prevent the overtreatment of PCa patients.

An understanding of many of the key molecules in the invasion and metastasis cascade is currently being formed, however, substantial gaps in our knowledge remain^5–8^. The six-transmembrane epithelial antigen of the prostate (STEAP) family contains four members and shares significant sequence homology with FRE metalloreductases in yeast at the C-terminus, and with bacterial and archaeal metalloreductases F_420_:NADPH-oxidoreductases (FNO) and human NADPH-oxidoreductase (NOX) at the N-terminus^9,10^. The N-terminal (apart from STEAP1) also contains a Rossman fold motif, which is thought to be vital for binding nucleotides such as flavin adenine dinucleotide (FAD). The six-transmembrane (6TM) domain, a heme-binding domain, is present in each protein and is also known as the apoptosis, cancer and redox associated transmembrane (ACRATA) domain^10^. This family is generally localised to the plasma membrane, golgi and trans-golgi network^11,12^. Our current functional understanding of this protein family in mammalian cells is limited; proteins containing the 6TM domain often serve as ion channels at cell junctions and due to the significant sequence homology with various metalloreductases, it has been suggested that the STEAP family may play a role in iron and copper reduction^9,11^. STEAP2, 3 and 4 expression has been shown to increase iron and copper uptake and promote reduction of iron and copper *in vitro*^9^.

A body of literature demonstrates altered STEAP gene expression patterns within several cancers. While this information presents the STEAP family members as important targets for a variety of diseases, our studies specifically focus on STEAP2, which appears to be specifically overexpressed in aggressive PCa^12–14^. We have recently described STEAP2 as overexpressed in PCa cells when compared to a normal prostate cell line and this was validated in a small pilot cohort of human tissues^14^. In the present study, we utilised two PCa cell lines, PC3 and LNCaP due to the fact they both originate from metastatic tumours and their high STEAP2 protein expression levels. The LNCaP cells express STEAP2 to a higher degree when compared to the PC3 cells, although the reason for this is, as yet, unknown^14^. Although both cells lines were originally derived from PCa metastases, they are technically distinct. The LNCaP cell line, which was derived from the supraclavicular lymph node by Horoszewicz in 1980, has been shown to express both androgen receptor (AR) and prostate specific antigen (PSA) and is androgen sensitive due to a mutation in the AR gene allowing it to bind to other steroids^15,16^. On the other hand, PC3 cells which were derived from bone metastases, do not express AR or PSA and do not respond to androgens^17^. LNCaP cells appear to share common features with adenocarcinomas, the most common diagnosed form of PCa; whilst PC3 cells appear to be more characteristic of the more aggressive prostatic small cell neuroendocrine carcinoma^18^. Additionally, while both LNCaP and PC3 cells have been shown to possess similar expression of proliferation-associated markers, differences in angiogenic markers, pro-inflammatory cytokines, PCa markers and responses to environmental stresses have been observed^19^. These two cell lines were therefore selected as a model to evaluate the functional role of STEAP2 in the development of aggressive cancer traits.

Due to the overexpression of STEAP2, specifically in invasive PCa, we hypothesised that STEAP2 may play an integral mechanistic role in progression to advanced disease. Hence, the aim of the present study was to establish the functional role of STEAP2 in driving PCa progression and to determine whether reduction of protein expression, via siRNA treatment, could reverse the tumourigenic impact of this protein.

## Results

The aim of this study was to develop an understanding of STEAP2’s role in driving prostate tumourigenesis by knocking-down STEAP2 expression (KD) in PC3 and LNCaP cells and analysing the subsequent functional changes compared to wild type (WT) and scrambled siRNA treated (Sc) cells as controls. Additionally, STEAP2 expression and localisation patterns were analysed in 164 PCa samples by IHC and correlation with Gleason score was explored.

### STEAP2 Knockdown Reduces Proliferative Potential in PC3 Cells

**Figure 1:**
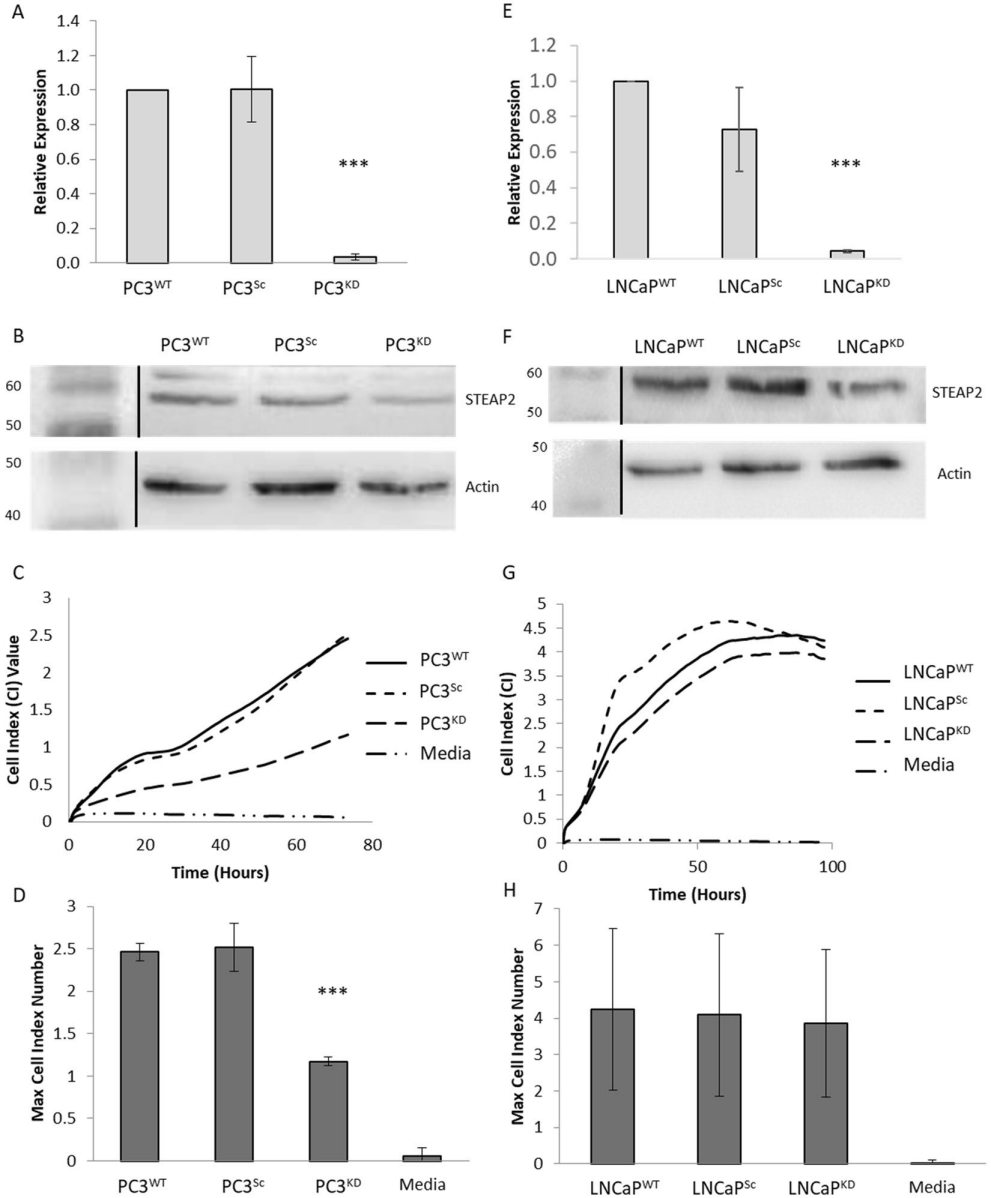
STEAP2 Knockdown results in a significant reduction in cell proliferation in PC3 cells. (**A**) qRT-PCR analysis for STEAP2 gene expression in control (PC3^WT^ and PC3^Sc^) and STEAP2-siRNA (PC3^KD^). (**B**) Western blot analysis for STEAP2 protein expression in control (PC3^WT^ and PC3^Sc^) and STEAP2-siRNA (PC3^KD^). (Loading control = Β-actin, Black lines represent where the western blot image has been edited for clarity). (**C**) Growth rate of the cells recorded in cell index (CI) values. PC3 cells monitored over a 72 h period and PC3^KD^ cells proliferate at a slower rate. (**D**) The maximum cell index values (i.e. final cell numbers) were significantly reduced in the PC3^KD^ cells compared to PC3^Sc^ control (***P < 0.001). (**E**) qRT-PCR analysis for STEAP2 gene expression in control (LNCaP^WT^ and LNCaP^Sc^) and STEAP2-siRNA (LNCaP^KD^). (**F**) Western blot analysis for STEAP2 protein expression in control (LNCaP^WT^ and LNCaP^Sc^) and STEAP2-siRNA (LNCaP^KD^). (Loading control = Β-actin, Black lines represent where the western blot image has been edited for clarity.) (**G**) LNCaP cells were monitored over a 96 h period and there was no difference observed between transfections. (**H**) The maximum cell index values were not altered in the LNCaP^KD^ cells compared to LNCaP^Sc^ control.

To investigate STEAP2’s role in the progression of PCa, expression was knocked down in PCa cell lines, PC3 and LNCaP. STEAP2 gene expression was knocked down by 97% and 95% in PC3^KD^ and LNCaP^KD^ cells respectively (Fig. 1A and E), this translated to a 40% and 50% respective reduction in protein levels over the experimental duration (Fig. 1B and F). To assess the effect of reducing STEAP2 expression on the proliferation of the PCa cells, iCELLigence analysis was carried out. PC3^KD^ cells exhibited a significant reduction (53%, p < 0.001) in the proliferation rate and maximum cell number when compared to PC3^Sc^ cells (Fig. 1C and D). Conversely, no significant difference was observed between the LNCaP^KD^ and LNCaP^Sc^ cells (Fig. 1G and H). To evaluate whether this effect on proliferation was due to cell cycle alterations in response to STEAP2 knockdown, cell cycle analysis was carried out on both PC3 and LNCaP cells treated with STEAP2 siRNA (Figure S1). For PC3 cells, no significant difference in the cell cycle profile was noted in response to STEAP2 knockdown, whilst in LNCaP^KD^ cells, a significant decrease in G0/G1 phase (p = 0.016) and a significant increase in G2/M phase (p = 0.019) was observed. However, as proliferative differences only arose in PC3^KD^ cells, this data suggest that an altered cell cycle profile was not subsequently responsible for their reduced proliferative capacity.

### STEAP2 knockdown Reduces Migratory Potential of PC3, and alters growth characteristics of LNCaP Cells

**Figure 2:**
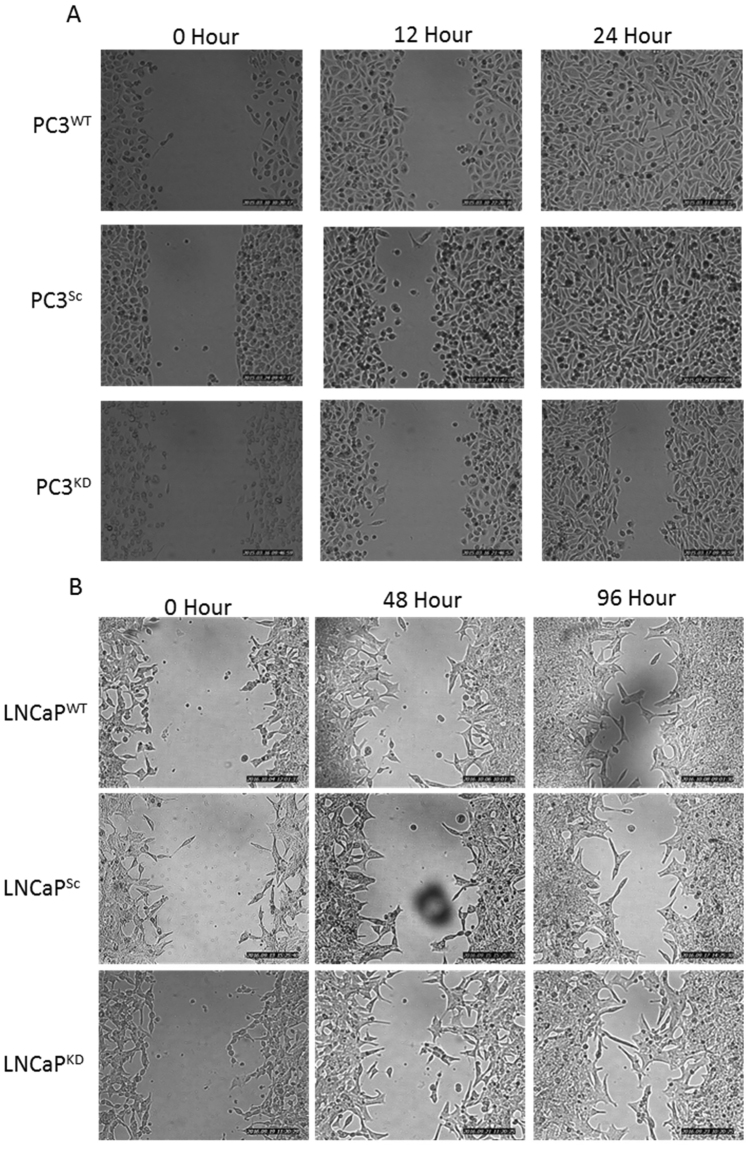
Decreasing STEAP2 expression significantly reduced the migratory capacity of the PC3 PCa cells. (**A**) PC3 cells shown as WT, Sc or KD imaged at 0, 12 and 24 h following the removal of the insert. The PC3^WT^ and PC3^Sc^ cells successfully closed the gap after 24 h; the gap remained open in the PC3^KD^ cells. (**B**) LNCaP cells shown as WT, Sc or KD imaged at 0, 48 and 96 hours following the removal of the insert. Decreasing the expression of STEAP2 in LNCaP cells had no significant effect of the migratory potential of the cells. The gap remained open in all samples after 96 h of observation.

**Figure 3:**
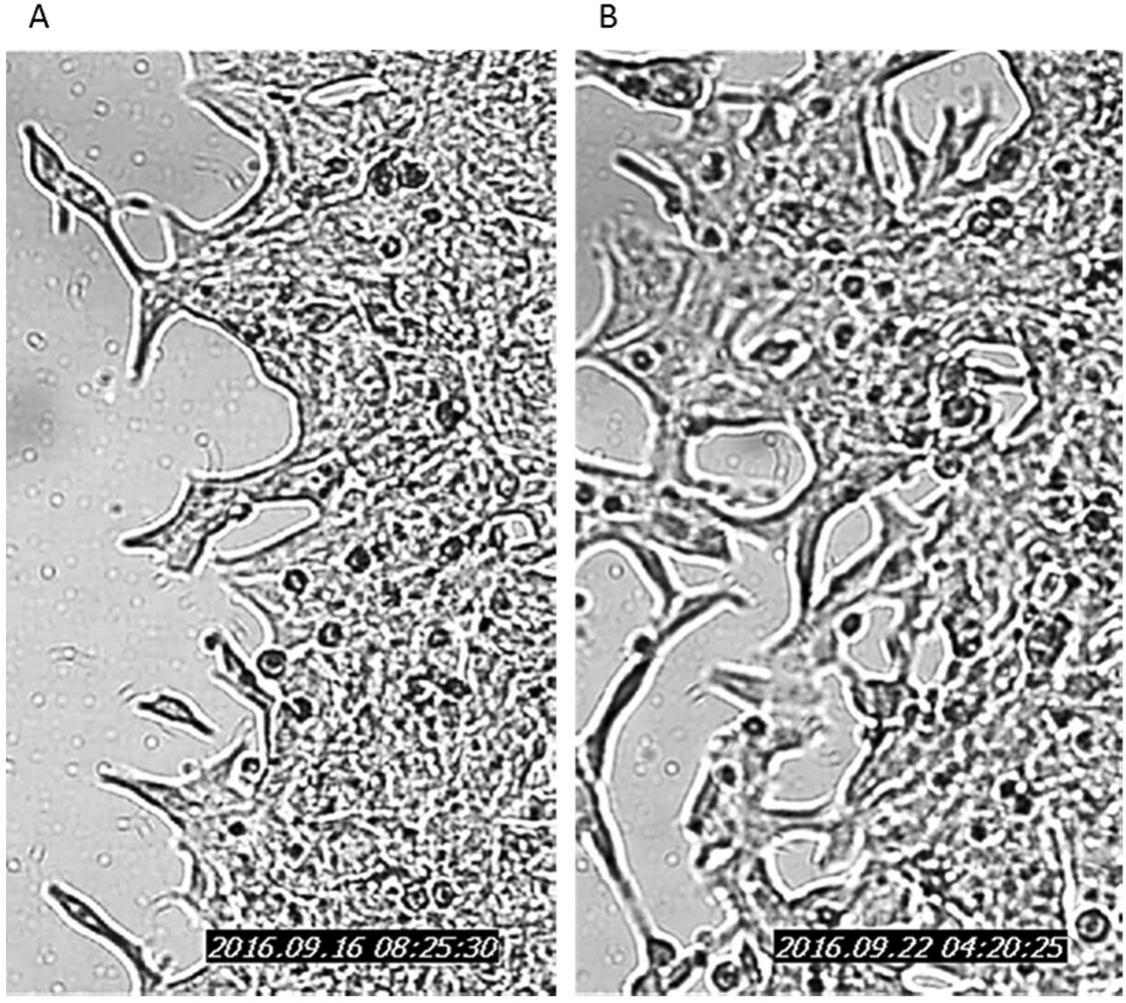
Representative images of the spread of LNCaP cells. (**A**) Scrambled siRNA treated LNCaP cells and (**B**) STEAP2 siRNA treated LNCaP cells. Both images were taken at the same magnification.

The ability of PC3 and LNCaP cells to migrate following the knock-down of STEAP2 was analysed using the IBIDI cell culture inserts. Representative images of time points 0, 12 and 24 h for PC3 cells and 0, 48 and 96 h for LNCaP cells are illustrated in Fig. 2. Both PC3^WT^ and PC3^Sc^ cells had closed the gap within 24 h following removal of the insert, however, in the PC3^KD^ cells, the gap remained open, demonstrating that reduction of STEAP2 expression in the PC3 cell line resulted in decreased migratory potential. LNCaP cells were examined over a 96 h period due to their slower and less uniform growth characteristics. The migration observed was to the same extent for all cells regardless of transfection status and complete closure was not achieved by the end of the 96 h time frame. The images taken of the LNCaP cells were analysed using ImageJ software and movies of the cells were generated (Figure S2 and S3). These movies revealed that the LNCaP^Sc^ cells move into the gap by growing on top of each other, which forces them into the empty space. In contrast, the movement of the LNCaP^KD^ cells was less haphazard, with the cells growing in a manner more closely resembling that of less invasive cells with contact inhibition, forming a monolayer rather than stacking on top of each other (Fig. 3). The images taken in Fig. 3 are the same magnification and time point, further demonstrating the changes in morphology in response to STEAP2 knockdown in LNCaP cells. The reduction of STEAP2 expression therefore had a notable consequence on the migration and growth characteristics of both LNCaP and PC3 cells.

### STEAP2 Knockdown Reduces Invasive Potential in both PC3 and LNCaP cells

**Figure 4:**
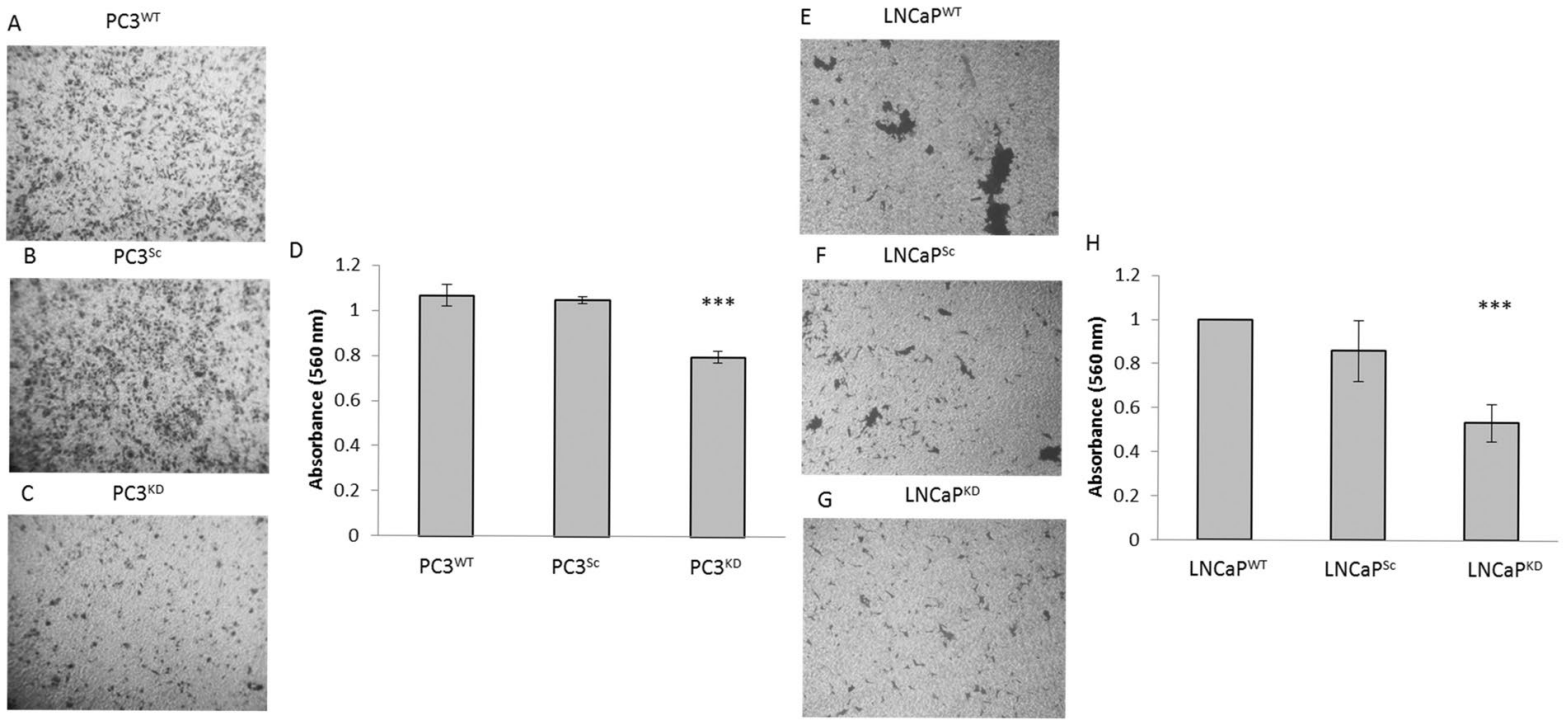
Decreasing STEAP2 expression significantly reduced the invasive potential of both the PC3 and LNCaP cells. Images of stained cells were taken to give a visual representation of invasion each panel represents (**A**) PC3^WT^, (**B**) PC3^Sc^, (**C**) PC3^KD^ cells and (**E**) LNCaP^WT^, (**F**) LNCaP^Sc^, (**G**) LNCaP^KD^ cells. (**D** and **H**) The stain was then extracted from the collagen matrix and the absorbance was measured to quantify the number of stained cells for PC3 cells and LNCaP cells respectively. The number of invasive cells was significantly lower in both the PC3^KD^ and LNCaP^KD^ cells (***P < 0.001 for both cell lines).

Cancer cells that have acquired invasive potential have increased ability to migrate into their surrounding area to establish distant metastases. Figure 4A–D illustrates that the invasive potential of PC3^KD^ cells was significantly reduced when compared to the PC3^Sc^ cells (36%, p < 0.001). Similarly, the LNCaP^KD^ cells had a significant 50% reduction of invasive potential when compared to the LNCaP^Sc^ cells (p < 0.001, Fig. 4E–H). Furthermore, while both the LNCaP^WT^ and LNCaP^Sc^ cells readily invaded through the membrane in large clusters, LNCaP^KD^ cells were more dispersed. This provides further evidence to support the increased contact inhibition exhibited by these cells following STEAP2 knockdown.

### STEAP2 Knockdown Alters Downstream Signalling in both PC3 and LNCaP cells

**Figure 5:**
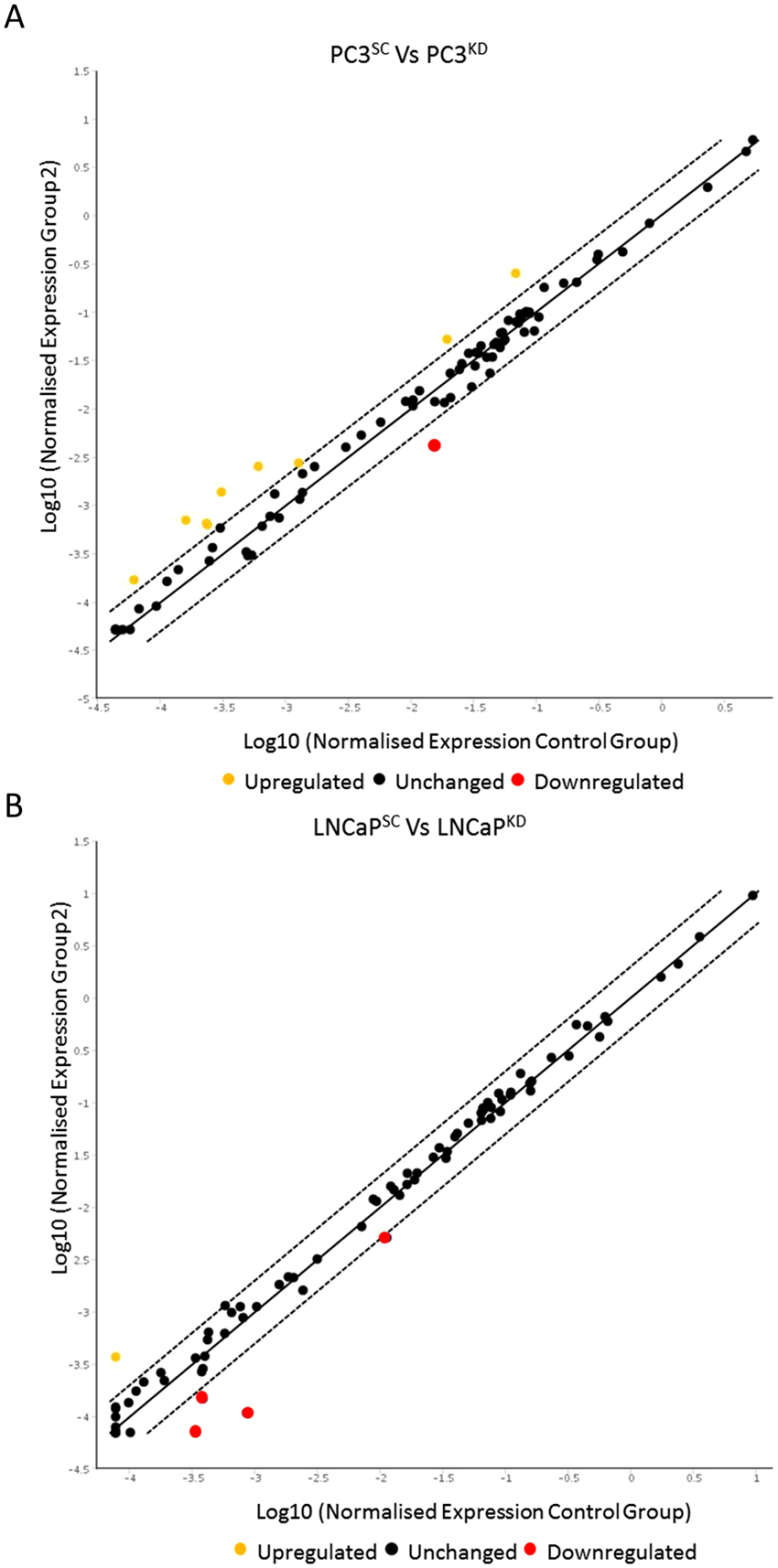
Normalised Expression of Tumour Metastasis Gene Array. The scatter plots were derived from comparison of the Scrambled siRNA treated cells (labelled as control) against the STEAP2-siRNA treated group (KD) in (**A**) PC3 cells or (**B**) LNCaP cells. The graphs show the number of up- and down-regulated genes in response to treatment depicted in yellow and red respectively.

Gene expression arrays were utilised to compare the expression changes of genes involved specifically in metastasis and invasion following STEAP2 knockdown. Figure 5 illustrates the results obtained from analysing 84 genes in the PC3 (A) and LNCAP (B) cells, a significant change was assigned when a ≥2-fold change in expression was recorded, p < 0.05. Genes demonstrating significant alterations in expression profiles in response to STEAP2 knockdown are summarised in Table 1. Some alterations in gene expression were only induced in scrambled siRNA treated cells, and these were excluded from the results as an artefact of the transfection process (*MMP13, FGFR4* and *MET*). qRT-PCR was utilised to validate the original array findings (Table 1). The alterations observed in response to STEAP2 knockdown were: *MMP3* (5-fold increase), *MMP10* (5-fold increase), *KISS1* (3-fold increase), *SERPINE1* (3-fold increase), *CD82* (2-fold increase) and *IL1β* (2-fold decrease) genes in PC3 cells and *MMP7* (2-fold decrease) and *CD82* (2-fold decrease) genes in LNCaP cells.

**Table 1:** Quantitative Real Time PCR Analysis of Array Results.

	Gene	Relative Change in Expression (according to gene array)	qRT-PCR data for WT cells	qRT-PCR data for KD cells
PC3	FGFR4	2.65	2.518*	3.283*
	IL1β	−3.64	0.917	0.564*
	KiSS1	2.79	1.699*	3.198*
	CD82	2.74	1.647*	2.695*
	MMP3	2.16	0.864	5.369*
	MMP10	4.47	2.739*	4.947*
	MMP13	4.19	4.781*	5.587*
	SERPINE1	2.70	1.421*	3.191*
LNCaP	CD82	−2.21	0.756	0.478*
	IGF1	−2.60	1.174	0.993
	MMP7	−4.82	0.881	0.434
	MET	4.79	3.523*	4.471

qRT-PCR was carried out on genes detected as altered in the tumour metastasis gene array and analysed in triplicate. Relative expression is depicted normalised to scrambled siRNA treated cells. Significant alterations *p < 0.05.

### STEAP2 Protein Expression is higher in Patient’s Tumour Tissue than in Matched Normal Samples and correlates with Gleason Score

**Figure 6:**
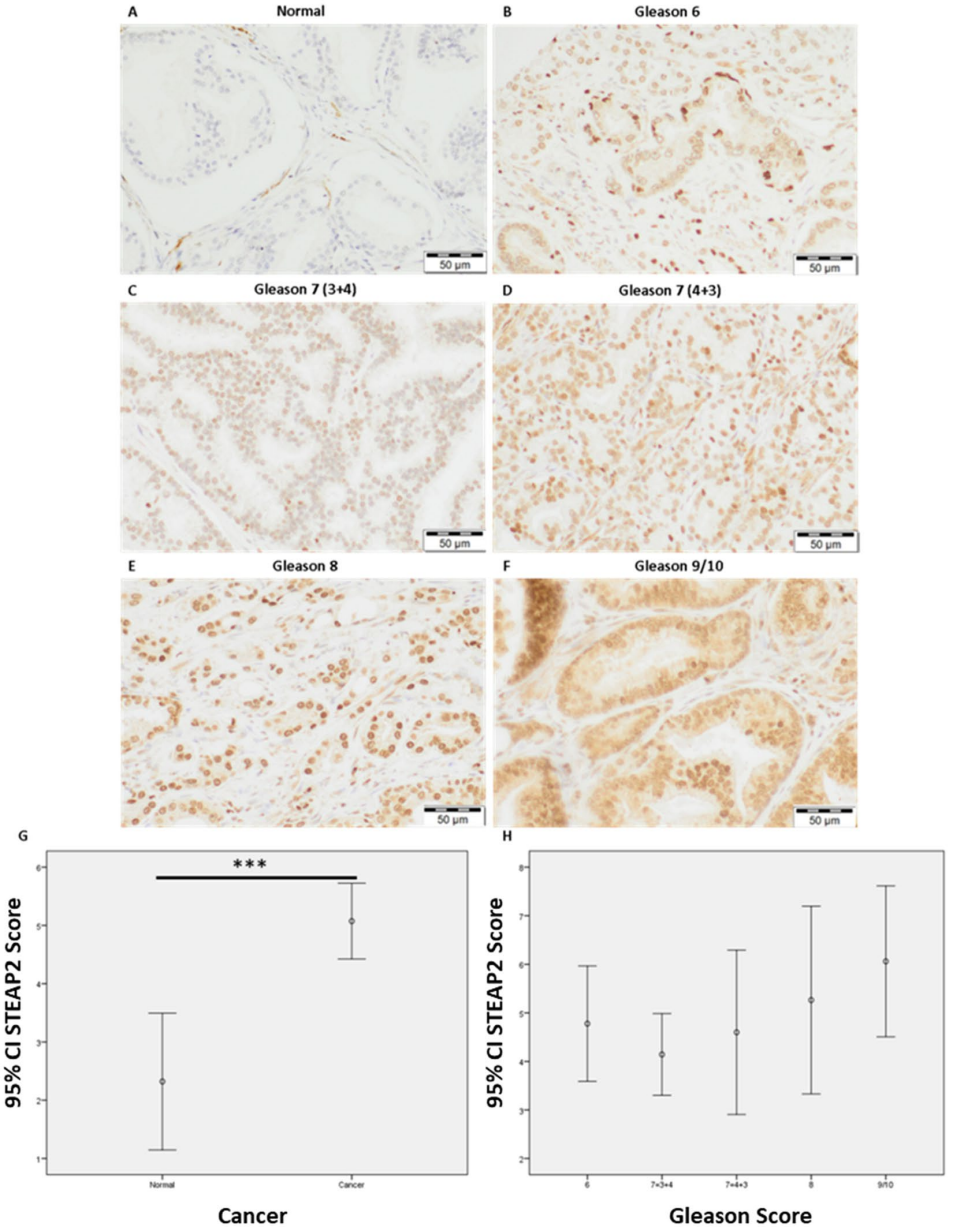
STEAP2 expression is higher in PCa specimens when compared to normal prostate tissue and increases with Gleason score. STEAP2 staining intensity was observed in (**A**) Normal patient samples and compared to (**B**) Gleason 6, (**C**) Gleason 7 (3 + 4), (**D**) Gleason 7 (4 + 3), (**E**) Gleason 8 and (**F**) Gleason 9/10. Staining intensity increased from normal to cancer samples and increased in a stepwise fashion from Gleason 6 to Gleason 9/10. (**G**) Significant difference in STEAP2 score observed between normal and cancer patients. (**H**) A significant positive correlation (r = 0.190) between Gleason score and STEAP2 score was determined using a Pearson correlation test. Significance denoted as ***p < 0.001.

The expression profile and localisation of STEAP2 was examined in normal and cancerous prostate specimens and correlation with tumour aggressiveness was evaluated by IHC. Representative images of STEAP2 staining in normal prostate and PCa tissue are presented in Fig. 6. STEAP2 expression is very low in normal prostate specimens, with only very light staining in the cytoplasm. STEAP2 was expressed to a significantly higher level in the PCa specimens when compared to the normal prostate specimens and had primarily nuclear localisation (p < 0.001, Fig. 6G). From Gleason 7(3 + 4 and 4 + 3) the intensity of STEAP2 stain increased in a stepwise fashion to Gleason 9/10 and a significant positive correlation was observed when STEAP2 score was compared against Gleason scores (r = 0.190, p = 0.049, Fig. 6H). These data suggest STEAP2 expression levels alone are not sufficient to distinguish between Gleason scores due to the similarities in staining, but that it would be a suitable biomarker to distinguish between normal prostate and PCa specimens.

## Discussion

The ability to discriminate between patients whose PCa will remain indolent and those that develop aggressive tumours is of great value to support patient risk stratification and aid clinical management of the disease. STEAP2 is elevated in PCa tissues, both *in vitro* and in clinical samples compared to normal prostate cells^12,14^. To determine the functional role of STEAP2 in prostate tumourigenesis, protein expression was reduced in PC3 and LNCaP cells and the subsequent impact analysed.

The hypothesis that increased STEAP2 expression results in a phenotype that aids in the progression of PCa was assessed by measuring the proliferation, migration and invasion of PC3 and LNCaP cells with reduced STEAP2 expression (KD) and comparing them to their wild type (WT) and scrambled siRNA treated (Sc) counterparts. The proliferation assay demonstrated that the growth of PC3^KD^ cells was decreased when compared to the PC3^Sc^ cells, supporting Whiteland *et al*.^14^; but this was not associated with altered cell cycle distribution. In contrast, the proliferative ability of LNCaP^KD^ was not altered, which contradicts the work of Wang *et al*. who showed a significant decrease of proliferation in LNCaP^KD^ cells^20^. This discrepancy could be due lack of comparison to the wild type or non-transfected LNCaP cells in the Wang *et al*. study. As demonstrated in the present study, the scrambled siRNA induces alterations in the cells that are not present in the wild type cells, therefore without this comparison; it is difficult to determine what is causing the alterations. Additionally, due to the high level of STEAP2 protein expression in the LNCaP cells and its long half-life; it is possible that the 50% reduction of protein expression observed in the present study had lesser impact on the pathways involved in proliferation. Further studies are required where these experiments are conducted on stably transfected LNCaP cells, using either shRNA, CRISPR or lentiviral knockdown, resulting in a more permanent reduction of STEAP2 expression.

The migratory potential of PC3^KD^ cells was shown to be significantly reduced when compared to the PC3^Sc^ cells, however, not in the LNCaP cells. The LNCaP cells are notoriously difficult to work with due to their reduced adherence and tendency to grow in clumps^21^. It was of interest to note that the growth pattern of the LNCaP cells were altered following STEAP2 knockdown. LNCaP^Sc^ and LNCaP^WT^ cells grow on top of each other rather than into the space created by the insert, which reflects the known growth characteristics of LNCaP cells^22^. However, the LNCaP^KD^ cells grew more readily as a monolayer with less stacking, suggesting that even a slight reduction of STEAP2 expression in the LNCaP cells results in partially reinstated contact inhibition, albeit not as profound as the effect on the PC3 cells. This observation indicated that STEAP2 may play a substantial role in the migration and growth characteristics of both cell lines. In the PC3^KD^ cells, the invasive potential was reduced by approximately 20%, while in the LNCaP^KD^ this was almost 50%. These data taken together therefore indicate a role for STEAP2 in the progression of PCa cells as the ability of the PC3 and LNCaP cells to both migrate and invade were altered when STEAP2 expression was reduced.

To further understand the downstream signalling targets influenced by STEAP2, a PCR gene array was utilised to evaluate its impact on 84 genes associated with metastasis. Eight genes were altered in PC3^KD^ cells and two in LNCaP^KD^ cells; *MMP13, MMP3, MMP10, FGFR4, KISS1, SERPINE1 CD82* and *IL1β* in the PC3 cells and *MMP7* and *CD82* in LNCaP cells.

*MMP3*, a member of the matrix metalloproteinase protein family, is involved in wound healing and degradation of the ECM during tissue remodelling and tumour metastasis^23^. *MMP3* overexpression has been reported in PCa as well as breast, lung and pancreatic cancer cells, tissues and mouse models, it is hypothesised to aid invasion and metastasis via degradation of the ECM^24–27^. It was therefore surprising that STEAP2 knockdown would result in a significant up-regulation *MMP3*. However, one clinical study on prostate tissues has reported a significantly decreased expression of MMP3 in PCa samples when compared to normal prostate and Benign Prostatic Hyperplasia (BPH) samples and this correlated with Gleason score^28^. Furthermore, most MMPs are produced and excreted from the cell in an inactive form, they are then activated extracellularly^29^. Increased expression may not correlate with increased pro-enzyme activation, thus further exploration of this enzyme activity status is required. *KiSS1* encodes the protein kisspeptin, a G-protein coupled receptor ligand for GPR54 and a metastasis suppressor in malignant melanoma and bladder cancer. Expression of *KiSS1 i*s reduced or lost in the more advanced cancers^30–33^. In the present study, reduced STEAP2 expression increases the expression of *KiSS1*, which suggests an inhibition of metastases (supported by the migration and invasion experiments). Thus, the relationship between STEAP2 and *KiSS1* may be an important signalling pathway responsible for controlling the metastatic potential of PCa cells. *SERPINE1*, which encodes the protein plasminogen activator inhibitor 1 (PAI-1), a serine protease inhibitor, was significantly overexpressed in response to STEAP2 knockdown. PAI-1 inhibits tissue plasminogen activator (tPA) and Urokinase (uPA), both of which are responsible for the cleavage of plasminogen to plasmin which goes on to mediate the degradation of the ECM in combination with MMPs, therefore facilitating invasion and metastatic spread^34^. This indicates an inhibition of invasion, which is supported by the experiments presented here, additionally, many studies have shown that PAI-1 prevents invasion of cancer cells by inhibiting uPA protease activity^35^. CD82, a tumour suppressor, was significantly overexpressed in the PC3^KD^ cells, but significantly decreased in LNCaP^KD^ cells. CD82 has been reported to be significantly overexpressed in BPH associated with cancer when compared to BPH not linked to cancer and thus CD82 overexpression may restrict PCa development while its loss may predispose the patient to more aggressive disease^36^. This therefore suggests that overexpression of STEAP2 and loss of CD82 both lead to aggressive PCa. The only down-regulation in PC3^KD^ cells was *IL1β*, an inflammatory cytokine important in mediating immune responses. IL1β plays an important role in the progression of PCa^37,38^. Recently, Liu *et al*. showed that IL1β was overexpressed in PCa and correlated with Gleason score^38^. Further to this, the group also demonstrated that overexpression of IL1β in non-metastatic PCa cells promoted their growth in the bone, while knockdown in metastatic cells inhibited bone progression, which is a particularly important observation given the down regulation noted here in PC3^KD^ cells^38^.

In the LNCaP^KD^ cells *MMP7* was significantly decreased when compared to the LNCaP^Sc^ cells. MMP7 is the smallest MMP family member and is overexpressed in many cancers including those of the bladder, colorectal, gastric and pancreatic^39^. Expression of MMP7 in mice promotes tumour development whereas reduction of MMP7 expression diminishes tumour incidence, although the exact mechanism is yet to be defined^40,41^. This protein is associated with increasing the invasive potential of cancer both *in vitro* and *in vivo* and is therefore important in the progression of aggressive disease^39^. This theory is supported by observations in the present study where a significant reduction of MMP7 expression in response to STEAP2 knockdown was also associated with reduced invasive capacity of the LNCaP^KD^ cells.

There was a much more pronounced effect on the expression of genes involved in the tumour metastasis pathway in the PC3^KD^ cells than the LNCaP^KD^ cells and there were no matched gene expression changes in the two cell lines. This is unsurprising given the different tissue origins of these cell lines and that their expression profiles represent two distinct PCa cell lineages^19^. The protein expression of STEAP2 in LNCaP cells is higher than in PC3 cells; given that a similar percentage of protein knockdown were achieved in both cell lines, the absolute levels of STEAP2 in the LNCaP cells following knockdown were therefore higher than in PC3, which could account for the greater transcriptional change noted in PC3 cells. Nonetheless, the reduction of STEAP2 protein expression has led to similar phenotypic changes in both cell lines; namely alterations in migration and a reduction in the invasive potential. This suggests that STEAP2 plays a significant role in such processes that are important in driving the development of aggressive PCa.

To support the functional cell analysis, the expression profile of STEAP2 in PCa was evaluated using IHC. This demonstrated a significant difference in STEAP2 staining intensity between normal prostate and PCa specimens, with a significant positive correlation between STEAP2 intensity and Gleason score. These data correspond with previous studies that have shown a significant increase of STEAP2 expression in cancer compared to normal samples in smaller patient cohorts^12,14^. One study on a small cohort of patients was unable to find such an association between the expression of STEAP2 in PCa tissue samples and Gleason scores, but it is possible that this was due to the limited patient cohort size^20^. Additionally, the spread of specimens is unknown as the author states that the samples were either < Gleason 7 (n = 22) or ≥ Gleason 7 (n = 45). Although a significant correlation was noted in the present study, it remains that the ANOVA analysis recorded no significant differences between groups. A larger patient cohort may assist in more clearly defining these differences, but nonetheless the present study presents a promising case for STEAP2 as a supporting prognostic biomarker for aggressive PCa.

This investigation has demonstrated that reducing STEAP2 expression in PCa cells significantly reduces their ability to proliferate, migrate and invade into their local surroundings. Although no significant difference in proliferation was observed in LNCaP cells in response to STEAP2 knockdown, a distinct difference in migratory behaviour, along with a significant reduction in invasive potential was observed in the LNCaP^KD^ cells, indicating that STEAP2 may be an interesting potential drug target in the future. Further to the *in vitro* study, STEAP2 expression and localisation were examined in a cohort of 164 PCa tissues; STEAP2 expression was increased in tumour tissue when compared to normal tissue and this significantly correlated with Gleason score. This gives rise to the possibility of utilising STEAP2 as a prognostic tool to determine the potential of PCa to progress to advanced disease.

## Methods

### Cell Culture

Authenticated PC3 and LNCaP cells purchased from American Type Culture Collection (ATCC, LGC Standard) were maintained in RPMI 1640 media (Gibco, Life Technologies, UK) supplemented with 10% fetal bovine serum (FBS), 2 mM glutamine, and 100 U/ml penicillin/streptomycin (Gibco, Life Technologies, UK) at 37 °C and 5% CO_2_.

### siRNA Transfection

STEAP2 expression was reduced using siRNA S48991, a non-specific siRNA (4390843) was used as a control (Life Technologies, UK) and Lipofectamine RNAiMAX (Life Technologies, UK) was used according to manufacturer’s instructions. PC3 cells were incubated with the siRNA complex for 72 h. LNCaP cells were reverse transfected with the siRNA complex for 120 h.

### Western Blot

Protein was extracted from PC3 and LNCaP cells using RIPA buffer (Sigma, UK) supplemented with protease inhibitor cocktail (1%, Sigma, UK). Protein (30 µg) was resolved using 10% sodium dodecyl sulphate polyacrylamide gel electrophoresis gels at 120 V for 90 minutes and transferred to polyvinylidene fluoride (PVDF) membrane at 400 mA for 90 minutes at 4 °C. Membranes were blocked using 5% milk in Tris Buffered Saline plus 1% Tween 20 (TBST) and incubated with STEAP2 antibody (1:1000, rabbit IgG, polyclonal, Sigma, UK) or β-actin antibody (1:1000, rabbit IgG, monoclonal, Cell Signalling, UK) overnight at 4 °C, antibodies were prepared in blocking buffer. Secondary antibodies (1:1000, Anti-rabbit IgG, Abcam, UK) were applied for 1 hour at room temperature. Images of the bands were visualised using Enhanced Chemiluminescence (ECL) reagent (Thermoscientific, UK).

### Quantitative Real Time PCR (qRT-PCR)

Total RNA was extracted from cells using the RNeasy extraction kit (Qiagen) following manufacturers instructions. cDNA was prepared using Retroscript reverse transcription kit (Ambion, Thermoscientific) according to manufacturer’s instructions. Pre-validated STEAP2 primers (NM_152999), along with primers for two housekeeping genes, β-actin (NM_001101) and Beta-2 microglobulin (B2M) (NM_004048), plus 2x precisionPLUS mastermix were obtained from PrimerDesign and qRT-PCR was carried out following the manufacturer’s instructions.

### Functional Assays

#### Proliferation

Experiments were carried out using the iCELLigence (ACEA Bioscience) according to the manufacturer’s instructions. Impedance was measured every minute for the first 2 h, then every hour for 72 h and displayed as arbitrary cell index (CI) values which represent the electrical impedance that results from proliferation (n = 3).

#### Cell Cycle

Cells were treated with SYTOX green (LitronLabs, USA) according to manufacturer’s instructions. As a positive control, cells were treated with 7.5 µM methyl methanesulfonate (MMS) for 23 hours to induce a G2 block. Samples were run using the BD FACS Aria flow cytometer (BD Biosciences, USA) (n = 3).

#### Migration

WT, SC and KD PC3 and LNCaP cells were seeded into the wells of the IBIDI cell culture inserts (IBIDI, Germany) and adhered for 24 h (PC3) or 48 h (LNCaP). Media and inserts were removed following incubation and fresh media applied. The time taken to close the gap created was monitored using a JuLi™ microscope over 24 h (PC3) or 96 h (LNCaP) (n = 3).

#### Invasion

Cells were serum starved for 24 h, harvested and re-suspended in serum-free media. The collagen inserts (ECM551, Merck Millipore, Germany) were rehydrated and 250 µl of cell suspension was applied. Serum-containing media (500 µl) was added to the bottom of the well and the inserts were incubated for 24 h (PC3) and 48 h (LNCaP) at 37 °C and 5% CO_2_. The cells that had invaded the collagen layer were subsequently stained and imaged according to manufacturer’s instructions (n = 3).

### Tumour Metastasis RT^2^ Profiler PCR Array

RNA (1 µg) was converted to cDNA using the RT^2^ First Strand Kit (Qiagen) and applied to the Tumour Metastasis RT^2^ Profiler PCR Array according to the manufacturer’s instructions. Genes that demonstrated significantly altered expression levels were verified by qRT-PCR analysis (n = 3). Primers were obtained from PrimerDesign: MMP 3 (NM_002422), MMP10 (NM_002425), MMP13 (NM_002427), FGFR4 (NM_002011), IL1β (NM_000576), KiSS1 (NM_002256), SERPINE1 (NM_000602), CD82 (NM_002231), IGF1 (NM_00111128), MET (NM_000245) and MMP7 (NM_002423).

### Immunohistochemistry

Pca Tissue Microarrays (TMAs), were custom built and provided by Wales Cancer Bank, Cardiff, UK following successful application to the biobank for the tissue samples to conduct the associated study. The Wales Cancer Bank is licensed by the Human Tissue Authority (licence 12107) to store human tissue for research, which has been obtained from patients providing informed consent. Wales Cancer Bank holds ethical approval from Wales Research Ethics Committee 3 to act as a research tissue biobank to both collect and issue biomaterials for cancer related research, where successful peer-reviewed applications have been approved. All methods applied in this report were therefore carried out in accordance with relevant guidelines and regulations in place for Wales Cancer Bank related studies. TMAs were constructed by Wales Cancer Bank from 164 PCa specimens with a range of Gleason scores. Three TMAs were generated in total containing: TMA1 – 68 cancer and 16 normal samples, TMA2 – 56 cancer and 14 normal samples and TMA3 – 40 cancer and 6 normal samples (normal samples were histologically normal tissues taken from patients with PCa). Patient information was provided in an anonymised fashion, including Gleason score, PSA values, age, death and relapse (Table 2). Formalin fixed, paraffin embedded sections were mounted on FLEX IHC slides, baked for 1 h at 60 °C and processed on a Benchmark XT automated staining system (Ventana ULTRA). Antigen retrieval was carried out for 32 minutes using cell conditioning buffer 1 (CC1), followed by pre-peroxidase inhibitor for 4 minutes at 36 °C. STEAP2 Primary antibody(1:50, rabbit,IgG, polyclonal, Abcam) was applied for 28 m at 36 °C. OV HQ Universal linker containing secondary antibody was applied (8 mins), OV- HPR multimer was applied (8 mins). OV DAB and H_2_O_2_ was applied (8 mins) before being incubated in copper (4 mins) followed by OV AMP multimer (4–8 mins). The slides were then counterstained with Haematoxylin for 8 mins. TMAs were scored by two individuals, blind to patient details. To ensure the control tissue used was histologically normal, with no possible infiltration of tumour, control cores were carefully evaluated in parallel H&E stained sections of each TMA by a consultant histopathologist. Additionally, as multiple sections were cut per TMA block to allow for all test antibodies to be analysed, H&E sections were generated from the first and last sections to ensure the cores scored were appropriately graded by the same consultant histopathologist. Samples with insufficient staining, poor quality of stain, compromised cores (e.g. rolled or partially missing) or lack of tumour cells in the sample were discarded from analysis. The test antibodies were optimised using positive control tissue, which was also run alongside all subsequent experiments to ensure the staining was appropriate for quality control purposes. Protein analysis was based on the intensity of the highest stain observed (scored 0 = none, 1 = low, 2 = medium and 3 = high) and the percentage of cells stained with this intensity (scored 0 = none, 1 = 1–25%, 2 = 26–50%. 3 = 51–75% and 4 = 76–100%). The final score was then obtained by multiplying the intensity and percentage scores together and were subsequently defined as: 0–2 = Low, 3–6 = Medium and 8–12 = High.

**Table 2:** Patient information relating to the prostate cancer specimens provided by WCB.

Gleason Score	No. of Tissue Samples	Age Range (Years)	PSA Range (ng/ml)	No. of Deaths/Relapse
Normal prostate tissue	28	48–75	2.8–19.6	2
6	18	49–71	2.2–19.6	2
3 + 4 = 7	28	49–74	4.5–22.5	3
4 + 3 = 7	10	54–75	2.2–18	1
8	19	48–72	3.1–31.7	6
9 and 10	33	43–84	2.2–160	14
Total	136	43–84	2.2–160	28

Number of tissue samples available for each STEAP2 analysis, along with age range, PSA range and number of death and/or relapse cases for patients falling into each Gleason score category. (“Normal” samples were from histologically normal areas of tissue from prostate cancer patients).

### Statistical Analysis

Statistical analysis was performed using IBM SPSS Statistics version 22. All comparisons to the KD samples were performed using a two-tailed, independent samples t-test. For IHC analysis, the following parametric tests were utilised; Pearson test to assess the correlation between STEAP2 and Gleason score and one-way ANOVA to determine differences between means. A p-value of <0.05 was considered significant.

### Data Availability

The datasets generated during and/or analysed during the current study are available from the corresponding author on reasonable request.

## Electronic supplementary material

Supplementary Dataset 1

Movie showing the migration of LNCaP cells treated with Scrambled siRNA.

Movie showing the migration of LNCaP cells treated with STEAP2 siRNA.
