# Supplementary material for: STEAP2 Knockdown Reduces the Invasive Potential of Prostate Cancer Cells
Source: Sci Rep. 2018 Apr 19;8:6252. doi: 10.1038/s41598-018-24655-x (PMC5908900; doi:10.1038/s41598-018-24655-x)
Supplement: Supplementary file 1 — Supplementary Dataset 1 [file 41598_2018_24655_MOESM1_ESM.docx]

**STEAP2 Knockdown Reduces the Invasive Potential of Prostate Cancer Cells**

Stephanie EA Burnell^1^, Samantha Spencer-Harty^2^, Suzie Howarth^3^, Owen Bodger^1^, Howard Kynaston^4^, Claire Morgan^1^, Shareen H Doak^1*^

^1^Institute of Life Science, Swansea University Medical School, Singleton Park, Swansea, SA2 8PP, Wales, UK
^2^Cellular Pathology, Abertawe Bro Morgannwg University Health Board, Singleton Hospital, Sketty Lane, Sketty, Swansea, SA2 8QA
^3^Histopathology, Abertawe Bro Morgannwg University Health Board, Morriston Hospital, Heol Maes Eglwys, Morriston, Swansea, SA6 6NL
^4^Cardiff School of Medicine, Cardiff University, Heath Park, Cardiff, CF14 4XN, Wales, UK

*Corresponding Author: Shareen H Doak –

Tel – 0044 1792 295388

Email - [S.H.Doak@swansea.ac.uk](mailto:S.H.Doak@swansea.ac.uk)


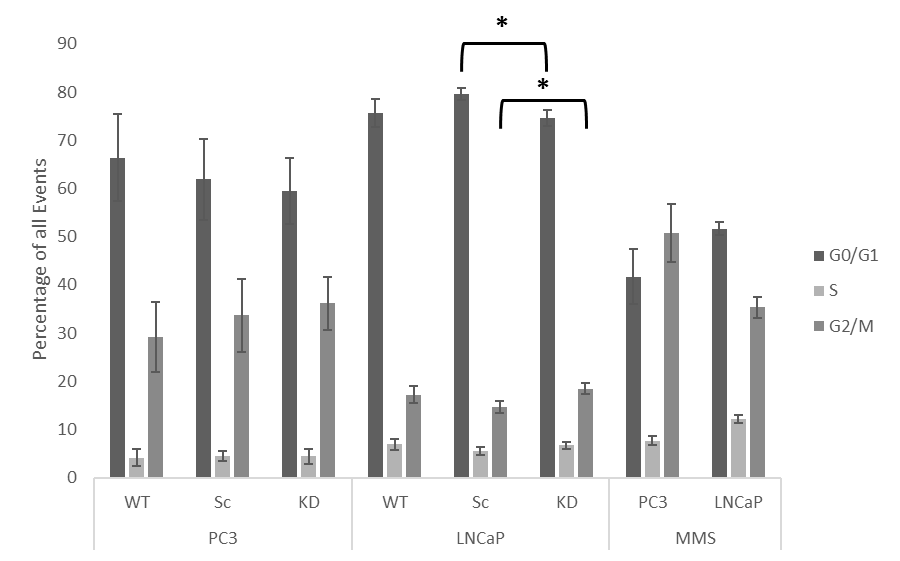


**Supplementary Fig. 1: Cell cycle profile analysis of STEAP2 siRNA treatment in PC3 and LNCaP cells.** The cells were serum starved for 48 hours to induce cell cycle synchronisation prior to siRNA treatment. Cells were treated with siRNA for 72 hours (PC3 cells) and 120 Hours (LNCaP cells) before cell cycle analysis was carried out. There was no significant change in any phase of the cell cycle upon STEAP2 siRNA treatment of the PC3 cells, however, there was a significant decrease in G0/G1 phase (p=0.016) and a significant increase in G2/M phase (p=0.019) upon STEAP2 siRNA treatment of the LNCaP cells (Significance denoted by *). The positive control for cell cycle analysis was a G2 block induced by 7.5 µM methyl methanesulfonate (MMS) treatment (n=3).
